# 899. Multi-modal Pediatric Education in an Adult Hospital

**DOI:** 10.1093/jbcr/irag033.395

**Published:** 2026-04-06

**Authors:** Ginny Figel, Abby L Hrabovsky, David Wingfield, Celeste Adan, Rahima Ladak

**Affiliations:** Walter L Ingram Burn Center, Grady Memorial Hospital, Atlanta, GA; Walter L Ingram Burn Center, Grady Memorial Hospital, Atlanta, GA; Grady Memorial Hospital, Atlanta, GA; Grady Health System, Atlanta, GA; Grady Memorial Hospital, Atlanta, GA

## Abstract

**Introduction:**

For decades, multidisciplinary pediatric care has required specialized knowledge and different competency training than adult care. In a large urban, ABA and ACS verified burn and Level 1 Trauma center which primarily handles adult care, the Burn Center is the only unit that admits pediatric patients for both burn and trauma care. Planned expansion of the unit with associated new staff hiring required an assessment and update of current pediatric education programs. Additionally, retention of a new RN Pediatric Specialist role within the Emergency Services Division allowed for the support of cross-service collaboration.

**Methods:**

A review of existing pediatric coursework was performed to identify gaps, and adult learning theory was utilized to optimize the educational environment. Standard lecture format has been updated to accommodate visual, auditory, and kinesthetic learning styles. Both high and low fidelity simulation is utilized from the beginning of the course to promote active learning. A didactic portion covers burn specific knowledge where staff and facilitators are encouraged to share their experience caring for pediatric patients on the unit, helping connect the “why” behind the education. Speakers include the unit Pharmacist, Social Worker, Child Life Specialist, and Behavioral Health Therapist, who educate on their specific roles during the care of pediatric patients. All courses have pre-class and post-class surveys; these were utilized by the authors to continue to modify the course.

**Results:**

32 staff have participated in this education program since the restructuring process. 100% of participants have reported being either “satisfied or very satisfied" by this program. Particularly, pre-assignment of the online modules ahead of time with the option for asynchronous learning has been a significant satisfier. Nursing simulation education was also a significant source of positive responses.

**Conclusions:**

Infrequent but highly stressful scenarios in medicine can be attributed to the intersection of subspecialty care; in this scenario burn care and pediatric care. Applying adult learning theory can potentially lower the cognitive load for learners; while this cohort of learners were not tested for knowledge retention, their self-reported satisfaction with the course suggests they identified the course as filling perceived gaps. Multi-modal education and consistent practice is paramount to keeping providers skilled in delivering the best care to this patient population.

**Applicability of Research to Practice:**

Focused, multimodal, and specialized education is important for nurses to be prepared to take care of all patients, especially as part of a team that cares for pediatric patients in an adult hospital. Engaging learners with instructors and each other creates a robust forum for problem solving and improved understanding.

**Funding for the study:**

N/A.

